# Antigen Uptake by Langerhans Cells Is Required for the Induction of Regulatory T Cells and the Acquisition of Tolerance During Epicutaneous Immunotherapy in OVA-Sensitized Mice

**DOI:** 10.3389/fimmu.2018.01951

**Published:** 2018-09-03

**Authors:** Vincent Dioszeghy, Lucie Mondoulet, Leo Laoubi, Véronique Dhelft, Camille Plaquet, Adeline Bouzereau, Christophe Dupont, Hugh Sampson

**Affiliations:** ^1^DBV Technologies, Montrouge, France; ^2^Department of Pediatric Gastroenterology Hepatology and Nutrition, Hôpital Necker Enfants Malades, Paris, France; ^3^DBV Technologies, New York, NY, United States

**Keywords:** food allergy, Epicutaneous immunotherapy, mechanisms, skin dendritic cells, Langerhans cells, regulatory T cells

## Abstract

The skin is a major immunologic organ that may induce protection, sensitization or tolerance. Epicutaneous immunotherapy (EPIT) has been proposed as an attractive strategy to actively treat food allergy and has been shown to induce tolerance in sensitized mice through the induction of Foxp3^+^ regulatory T cells (Tregs), especially CD62L^+^ Tregs. Among immune cells in the skin, dendritic cells are key players in antigen-specific immune activation or regulation. The role of different populations of skin DCs in tolerance induction remains to be elucidated. Using OVA-sensitized BALB/c mice, we demonstrated that the application of a patch containing OVA-A647 to the skin resulted in allergen uptake by Langerhans cells (LCs) and CD11b^+^ dermal cDC2 and subsequent migration into skin draining lymph nodes. These 2 populations induced Foxp3 expression in CD4^+^ cells *in vitro*. Only LCs induced LAP^+^ cells and CD62L^+^ Tregs. Using Langerin-eGFP-DTR mice, we analyzed the role of LCs in the mechanisms of tolerance induction by EPIT *in vivo*. Following complete depletion of LCs, a dramatic decrease in the number of OVA^+^ DCs and OVA^+^ CD11b^+^ dermal cDC2 was observed in skin draining lymph nodes 48 h after epicutaneous application. Likewise, 2 weeks of EPIT in non-depleted mice induced Foxp3^+^ Tregs, especially CD62L^+^, and LAP^+^ Tregs in skin draining lymph nodes and spleen, whereas no induction of Tregs was observed in LC-depleted mice. Following 8 weeks of treatment, EPIT-treated mice showed significant protection against anaphylaxis accompanied by a significant increase of Foxp3^+^ Tregs, especially CD62L^+^ Tregs, which was not seen in the absence of LCs. In summary, although both LCs and CD11b^+^ dermal cDC2s could induce regulatory T cells, the absence of LCs during EPIT impaired treatment efficacy, indicating their crucial role in skin-induced tolerance.

## Introduction

Food allergy is a growing public health concern and manifestations can be severe even life-threatening. Its prevalence increased dramatically during the last decades up to 10% in 2018 [1]. There is no approved cure other than strict avoidance of identified foods and availability of self-injectable epinephrine. Allergen-specific immunotherapy aiming at reduction of the sensitivity to an allergen is an attractive strategy to actively treat food allergy. Proposed imunological mechanisms include decreased allergen-specific IgE and increased IgG4 levels, reduced responses of effector CD4^+^ T cells, and induction of regulatory T cells (Tregs). Epicutaneous immunotherapy (EPIT) is a novel therapy that is currently under investigation. Safety and efficacy of Viaskin® Peanut in allergic patients has been studied in a recent phase 3 clinical program after positive results in a phase IIb trial (2).

The mechanisms of EPIT have been investigated in animal models of food allergy. In sensitized mice, EPIT with the corresponding allergen induced desensitization (3, 4), protected from allergic gut inflammation (5) and prevented food-induced anaphylaxis (6) and sensitization to new allergens (7). Tregs induced by EPIT are key: the protective effect of EPIT is abrogated by depletion of CD25^+^ cells and is transferred to sensitized mice by the transfer of Tregs (8). EPIT increased CD62L^−^ and CD62L^+^ Foxp3^+^ Tregs whereas other immunotherapies induced mainly CD62L^−^ Tregs in peanut sensitized mice (9). In a recent study, the sustained protection and bystander effect of EPIT was related to epigenetic modifications in this CD62L^+^ population of Tregs (10).

The unique immunologic features of the skin keratinocytes and antigen-presenting cells might explain the specific induction of this particular population of Tregs by EPIT. Among immune cells in the skin, dendritic cells (DCs) are key players in antigen-specific immune activation or regulation. Skin DCs can be divided into Langerhans cells (LCs) in the epidermis, the superficial layer of the skin, and a different subset of dermal DCs closely related to conventional DCs, which may themselves be divided into cDC1s and cDC2s, identified based on XCR1 and CD11b expression, respectively. It has been shown that cDC1s are more prone to induce a Th1 response and cross-present Ag to CD8^+^ cells, whereas cDC2s are more likely to induce a Th2 response. LCs activate and induce Th17 or Tregs depending on the environmental stimuli (11). In previous studies, we demonstrated rapid allergen uptake by dendritic cells through intact skin leading to down-regulation of allergen-specific responses in sensitized mice treated by EPIT (12). However, the role of the different populations of skin DCs in tolerance induction remains to be elucidated.

Using OVA-sensitized mice, we demonstrated that epicutaneous application of allergen resulted in uptake by LCs and CD11b^+^ dermal cDC2s. After migration into skin draining lymph nodes (sdLNs), both populations could induce Foxp3 expression in CD4^+^ T cells *in vitro*. However, only LCs induced LAP^+^ cells and CD62L^+^ Tregs, and the depletion of LCs significantly decreased the induction of Foxp3^+^ Tregs, especially CD62L^+^ and LAP^+^ Tregs in skin draining lymph nodes and spleen, compared to non-depleted mice. Moreover, the EPIT-induced protection against anaphylaxis was not seen in the absence of LCs. Thus, although both LCs and CD11b^+^ dermal cDC2 can induce regulatory T cells, the absence of LCs during EPIT reduced the effectiveness of the treatment, indicating their crucial role in skin-induced tolerance.

## Methods

### Mice

BALB/c mice were obtained from Charles River (Charles Rivers, Lyon, France). Langerin-DTR mice, obtained from Pr Malissen (Centre d'Immunologie de Marseille-Luminy, Aix Marseille Université, INSERM, CNRS UMR, Marseille, France), were back-crossed on a BALB/c background and maintained as breeding colonies at DBV-Technologies. All procedures were performed according to the European Community rules on animal care with permission and ethical approval # 7811 from the French Authorities. Mice were acclimated in the animal facility for 1 week prior to initiating any procedures. Each experiment was reproduced twice independently.

### Sensitization

Mice were sensitized on days 0 and 7 with 200 μl of a homogenous suspension of 10 μg of OVA (grade V, Sigma-Aldrich, France) and 2 mg of aluminum hydroxide (Imject® Alum, Thermo scientific, France) by subcutaneous administration on the back of the neck. Mice were then boosted intranasally on day 14 with OVA (10 μg).

### Measurement of allergen capture by skin DCs

For epicutaneous application, the skin of sensitized mice was shaved with electric clippers and then depilatory cream was applied 24 h before applying the epicutaneous patch. For studies of allergen capture and skin DCs migration, VIASKIN® with 100 μg OVA-alexa647 (V-OVA) were applied for indicated times on the back of mice and maintained with a non-occlusive linen. Mice were then sacrificed and skin and skin-draining lymph nodes (sdLN) were harvested.

### Skin and sdLN cell isolation

Using an insulin microneedle, 8 mm skin biopsies were injected 5–10 times with Liberase TL (250 μg/mL)-DNase (500 μg/mL) solution, placed in 1 ml of Liberase TL-DNase solution in a 24-well plate (dermal side down), and then incubated for 2 h at 37°C in a cell culture incubator (5% CO_2_). To stop the enzymatic action at the end of the incubation period, 500 μL of DNase working solution and 75 μl of 0.1 M EDTA were added. Tissues were placed in a Medicon tissue grinder (BD Biosciences) and mixed for 8 min in a Medimachine (BD Biosciences). Cell suspensions were filtered using 50 μm syringe filter (Filcon, BD Biosciences), washed and counted.

Skin draining lymph nodes were harvested in Petri Dishes containing 1 mL RPMI 1640. Then, 1 mL of Liberase TM (0.52 U/mL)/DNase I (50 μg/mL) in RPMI 1640 were added to each Petri Dish. Each LN was flushed using an insulin microneedle and incubated for 20 min at 37°C. Two hundred fifty microliter of solution of EDTA at 100 mM were added to each Petri Dish to stop the reaction. Lymph node cell suspensions were then obtained by subsequent tissue dissociation and filtration through a cell strainer (100 μm), washed and counted.

### *In vitro* tregs induction measurement

OVA^+^ migrating LCs and CD11b^+^ DCs were isolated from sdLN cells by magnetic sorting using a CD11c isolation kit (Miltenyi Biotec, Paris, France) followed by flow cytometry sorting of MHC-II^+^ CD11c^+^ OVA^+^ CD11b^+^ EPCAM^+^ and MHC-II^+^ CD11c^+^ OVA^+^ CD11b^+^ EPCAM^−^, respectively. As control DCs, MHC-II^+^ CD11c^+^ OVA^−^ CD11b^−^ EPCAM^−^ cells were also sorted from the CD11c positive fraction. CD4^+^ cells were sorted from the CD11c negative fraction using CD4 microbeads (Miltenyi Biotec, Paris, France). Each DC subset was then co-culture with CD4^+^ cells at ratio 1:5 in 200 μL RPMI 1640 supplemented with FCS, penicillin, streptomycin, and β-mercaptoethanol in 96 wells plate. After 6 days, cells were stained for analysis of Foxp3 or LAP Tregs and expression of CD62L.

### Epicutaneous immunotherapy

Sensitized mice were treated weekly with VIASKIN® containing 100 μg OVA for 48 h. Tregs induction was measured after 2 weeks of treatment in sdLN and spleen. For measurement of allergen-specific oral reactivity, sensitized mice treated for 8 weeks or not treated were boosted by oral gavage with 20 mg OVA every 3 days X's 6 before receiving an oral challenge with 50 mg OVA. Animal temperatures were measured before and during challenge using microchips (Plexx IPTT). Single cell suspensions of mouse spleen were obtained by tissue dissociation and filtration through a cell strainer for *in vitro* re-stimulation and cytokine production measurements previously described (ref).

### Langerhans cells depletion

Langerin-DTR mice were injected i.p. with 0.1 μg of diphteria toxin 5 days and 1 day before the first application of VIASKIN® and every 2 weeks 24 h before VIASKIN® application. This resulted in complete depletion of skin Langerhans cells throughout the treatment period, as verified by flow cytometry (data not shown).

### Flow cytometry

For DC analysis in the skin and sdLNs, cells were stained with different combinations of the following antibodies: MHC-II-Vioblue, CD11c-PerCPVio700, CD11b-VioGreen, CD11b-PerCPVio700, XCR1-Viobright-FITC, EPCAM-PE, CD86-PE-Vio770, CD86-APC-Vio770, PD-L2-PE-Vio770 (all from Miltenyi Biotec), CD11c-APC-Cy7 (BD Biosciences), XCR1-BV510 (Biolegend). Dead cells were excluded from analysis using Zombie dye (Biolegend) of appropriate color depending of the antibodies used.

For Tregs analysis, cells were stained with combinations of the following antibodies: anti-mouse CD4-FITC (Miltenyi), CD25-APC-Cy7, CD62L-PE-Cy7 (all from BD Biosciences, Le Pont de Claix, France), Latency-associated peptide (LAP)-PE and Foxp3-APC (from e-Bioscience, Paris, France), or control isotypes. Intracellular staining was performed after fixation and permeabilization, using Foxp3 Perm Kit (e-Bioscience, Paris, France). Dead cells were excluded from analysis using Zombie dye aqua (Biolegend).

Flow cytometry was performed on a MACSquant cytometer and analyzed using FlowJo software.

### Statistical analysis

The GraphPad Prism Software 5.0 (San Diego, CA, USA) was used for statistical analyses. Results are expressed as median with range. Statistical significance comparing different sets of mice were determined by Kruskal-Wallis test followed by Dunn's multiple comparisons test.

## Results

### Characterization of dendritic cells implicated in allergen uptake in OVA sensitized mice

**Figure 1:**
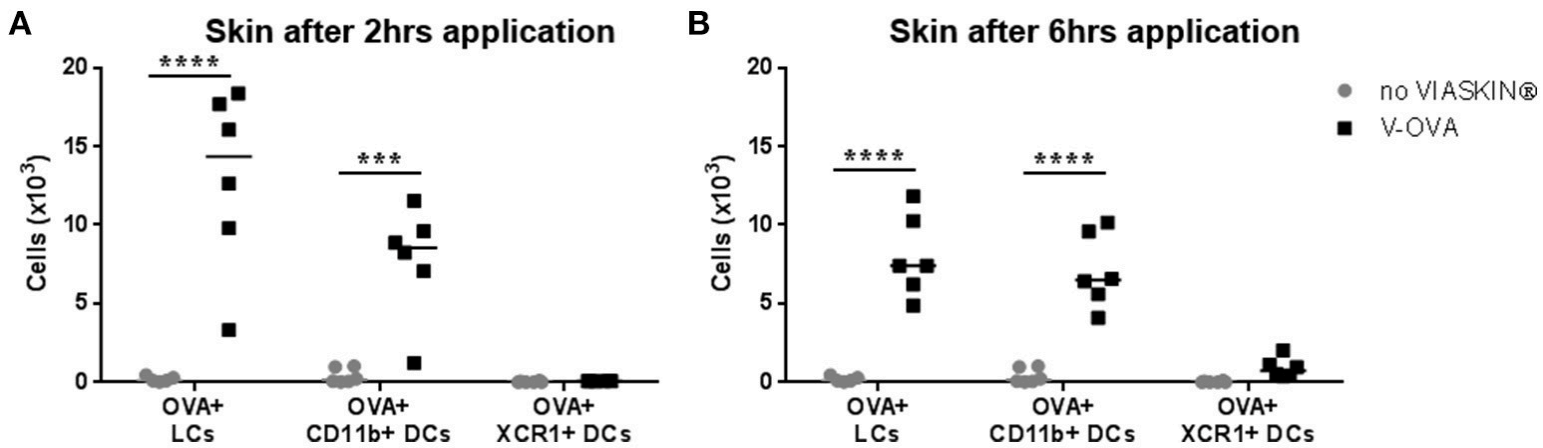
Allergen capture by skin dendritic cells after epicutaneous application. VIASKIN®-OVA-AF647 (V-OVA) was applied on the back of sensitized mice for 2 h **(A)** or 6 h **(B)**. Sensitized not treated mice were used as control (no VIASKIN®). Skin were then harvested and cells isolated and counted before immunostaining for flow cytometry analysis. In live single cells gate, migrating DCs were gated as MHC-IIhigh CD11c+ and Langerhans cells (LCs), CD11b^+^ DCs and XCR1^+^ DCs were identified as CD11b^+^EPCAM^+^XCR1^−^, CD11b^+^EPCAM-XCR1–, and CD11b-EPCAM-XCR1^+^, respectively. Proportion of OVA+ cells inside each population were measured and reported to number of cells. Each symbol represents a mice and bar represent median. Experiment was reproduced twice independently. ^***^p < 0.001; ^****^p < 0.0001 by 2-way ANOVA followed by Sidak's multiple comparisons test.

To determine which skin DCs take up allergen following epicutaneous application, sensitized mice were treated with VIASKIN®-OVA-AF647 for 2 or 6 h. Evaluating OVA^+^ DCs from each subset showed that both LCs and dermal CD11b^+^ DCs (DC2s) captured allergen efficiently after 2 h whereas XCR1^+^ DCs (DC1s) did not (Figure 1A). After 6 h of application, OVA^+^ LCs and OVA^+^ CD11b^+^ DCs were still present in the skin, but to a lesser extent suggesting that some cells had already migrated from skin. Extremely low levels of OVA^+^XCR1^+^ cells were detectable even after 6 h of application (Figure 1B).

**Figure 2:**
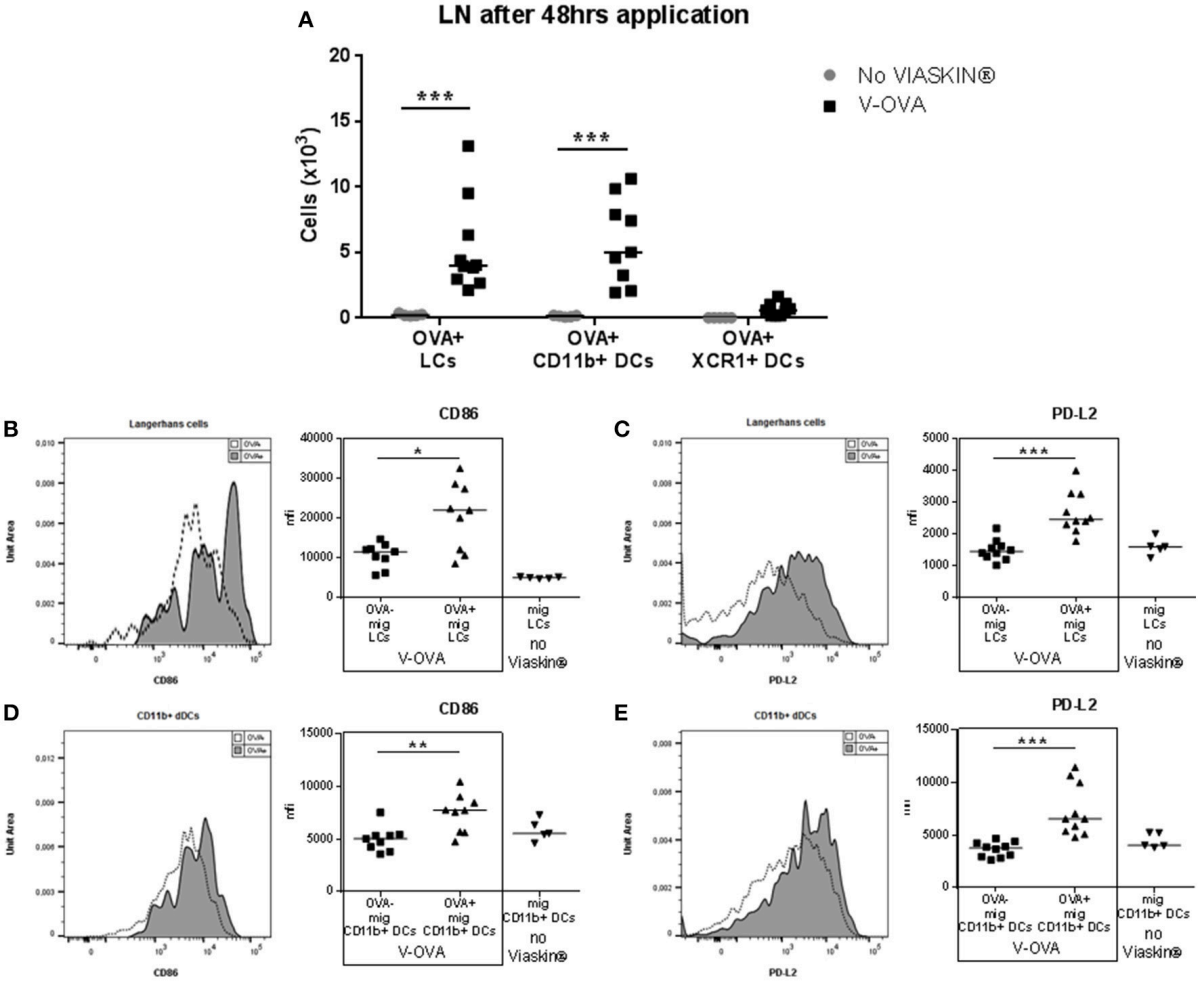
Migration toward draining lymph nodes of skin dendritic cells that capture allergen after epicutaneous application. VIASKIN®-OVA-AF647 (V-OVA) was applied on the back of sensitized mice for 48 h. Sensitized not treated mice were used as control (No VIASKIN®). sdLN were then harvested and cells isolated and counted before immunostaining for flow cytometry analysis. In live single cells gate, migrating DCs were gated as MHC-II^high^ CD11c^+^ and Langerhans cells (LCs), CD11b^+^ DCs and XCR1^+^ DCs were identified as CD11b^+^EPCAM^+^XCR1^−^, CD11b^+^EPCAM^−^XCR1^−^, CD11b^−^EPCAM^−^XCR1^+^, respectively. Proportion of OVA^+^ cells inside each population were measured and reported to number of cells **(A)**. Expression of activation marker CD86 **(B,D)** and PD-L2 **(C,E)** were analyzed in OVA^−^ and OVA^+^ LCs or in total migrating LCs in not treated mice **(B,C)** and in OVA^−^ and OVA^+^ CD11b^+^ DCs or in total migrating CD11b^+^ DCs in not treated mice **(D,E)**. Each symbol represents a mice and bar represent median. Experiment was reproduced twice independently. ^*^p < 0.05; ^**^p < 0.01; ^***^p < 0.001 by Kruskal-Wallis test followed by Dunn's multiple comparisons test.

In previous experiments, after application of VIASKIN®-OVA on intact skin of OVA-sensitized mice, skin dendritic cell migration toward sdLN peaked after 48 h (12). We then characterized the migration of skin dendritic cells into sdLNs after 48 h. Skin migrating DCs were gated as CD11c^+^ cells expressing high levels of MHC-II. Among skin migrating cells, the 3 subsets were gated using XCR1, CD11b, and EpCAM expression. As expected from results in the skin, OVA was detected in LCs and CD11b^+^ DCs, and to a lesser extent in XCR1^+^ DCs (Figure 2A). Skin and sdLN results strongly suggest that allergen uptake following epicutaneous application in sensitized mice is mediated by LCs and CD11b^+^ DCs. We then further analyzed their activation status by evaluating the expression of CD86 and PD-L2. OVA^+^ LCS and OVA^+^ CD11b^+^ DCs had significantly increased expression of CD86, indicating a higher activation status compared to OVA- migrating cells (Figures 2B,D, respectively). Both populations of OVA^+^ cells also expressed significantly higher levels of PD-L2 suggesting a potential tolerogenic capacity (Figures 2C,E). As a control for activation, skin DCs migrating to sdLN after application of VIASKIN®-OVA on tape-stripped skin expressed higher levels of CD86, but lower levels of PD-L2 (data not shown).

**Figure 3:**
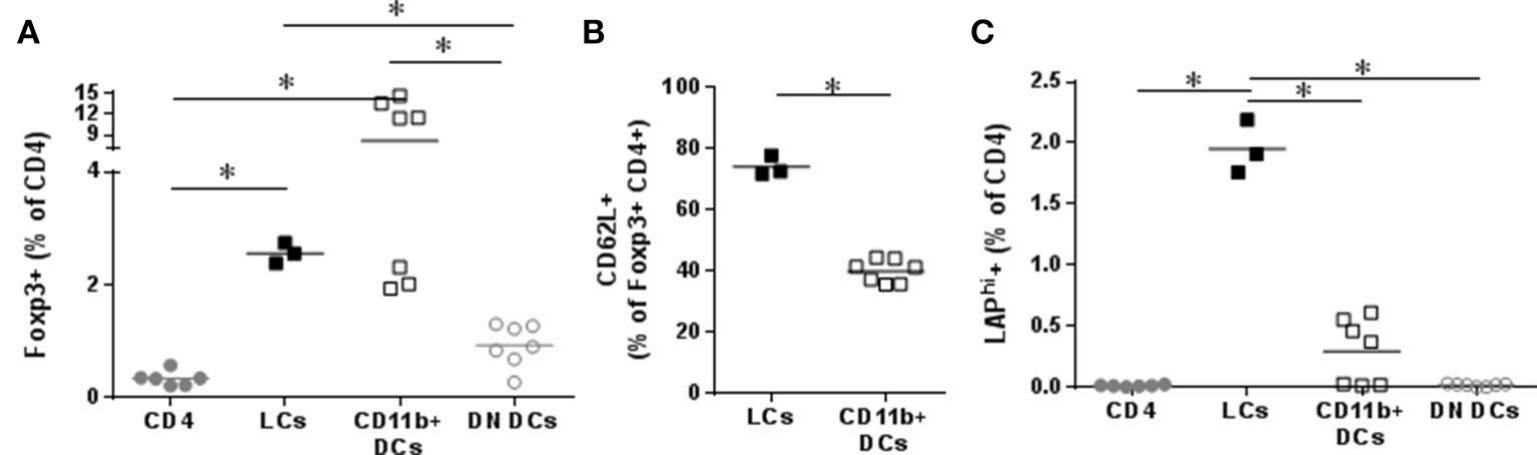
Capacity of skin migrating Dendritic Cells to induce Tregs. VIASKIN®-OVA-AF647 (V-OVA) was applied on the back of sensitized mice for 48 h. sdLN were then harvested and cells isolated and OVA^+^ Langerhans cells (LCs), OVA^+^ CD11b^+^ DCs and OVA^−^ EpCAM^−^ control DCs (DN DCs) were FACS sorted and cultured with CD4+ cells for 6 days. Proportion of CD25^+^Foxp3^+^ among CD4^+^ cells **(A)**, of CD62L^+^ among CD25^+^Foxp3^+^ **(B)** and of LAP^hi^ **(C)** among CD4 cells were analyzed. Each symbol represents a well and bar represent median from two independent experiments. *p < 0.05 by Kruskal-Wallis test followed by Dunn's multiple comparisons test.

To analyze their tolerogenic potential, we measured LC and DC capacity to induce CD4^+^ cells into regulatory T cells *in vitro*. OVA^+^ LCs and OVA^+^ CD11b^+^ DCs were FACS sorted and co-cultured with CD4 T cells. Both populations significantly increased Foxp3 in CD4 cells *in vitro* compared to CD4 cultured alone or in presence of control DCs (Figure 3A). We had previously shown the capacity of EPIT to induce CD62L^+^ Tregs in comparison to other forms of immunotherapy, e.g., oral and sublingual immunotherapy, and checked the proportion of CD62L in *in vitro* induced Tregs. OVA^+^ LCs induced higher levels of CD62L on Foxp3^+^CD4 cells compared to CD11b^+^ DCs (Figure 3B). Furthermore, only OVA^+^ LCs significantly increased LAP^+^ CD4 cells *in vitro* (Figure 3C) suggesting that LCs are the key players in Tregs induction during EPIT.

**Figure 4:**
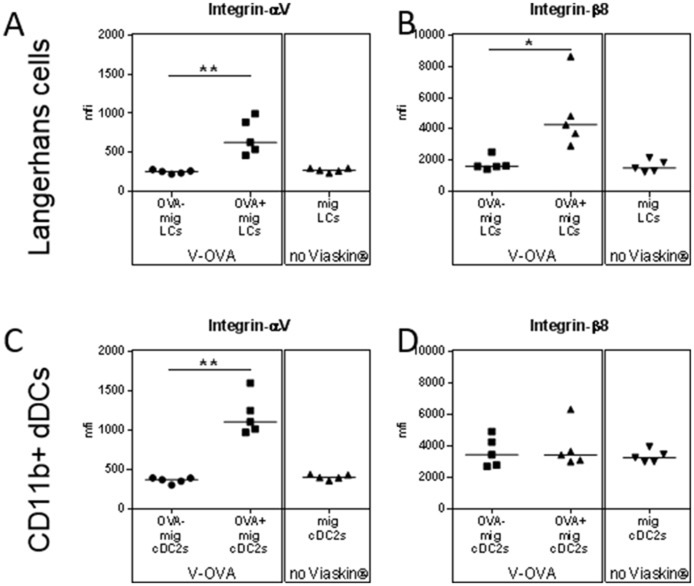
Capacity of skin migrating Dendritic Cells to induce Tregs is mediated by TGF-β. **(A–D)** VIASKIN®-OVA-AF647 was applied on the back of sensitized mice for 48 h. Sensitized not treated mice were used as control (No VIASKIN®). sdLN were then harvested and cells isolated and counted before immunostaining for flow cytometry analysis. Expression of integrin alphaV **(A,C)** and beta8 **(B,D)** were analyzed in OVA^−^ and OVA^+^ LCs or in total migrating LCs in not treated mice **(A,B)** and in OVA^−^ and OVA^+^ CD11b^+^ DCs or in total migrating CD11b^+^ DCs in not treated mice **(C,D)**. ^*^p < 0.05; ^**^p < 0.01; by Kruskal-Wallis test followed by Dunn's multiple comparisons test.

The generation of peripheral Tregs requires TGF-β (13). The ability of DCs to activate TGF-β from its “latent” precursor has been recently linked to the expression of specific integrin by DCs in gut, i.e., alphaV beta8 (14, 15). In sdLN, OVA^+^ skin migrating LCs and CD11b^+^ dDCs expressed higher level of integrin alphaV than OVA^−^ or control DCs (Figures 4A,C). OVA^+^ skin migrating LCs also expressed higher level of integrin beta8 compared to OVA^−^ or control LCs whereas CD11b^+^ dDCs did not (Figures 4B,D), suggesting a possible role of integrin beta8, and TGF-β in the capacity of migrating LCs to induce Tregs.

### Characterization of the allergen uptake in absence of LCs

**Figure 5:**
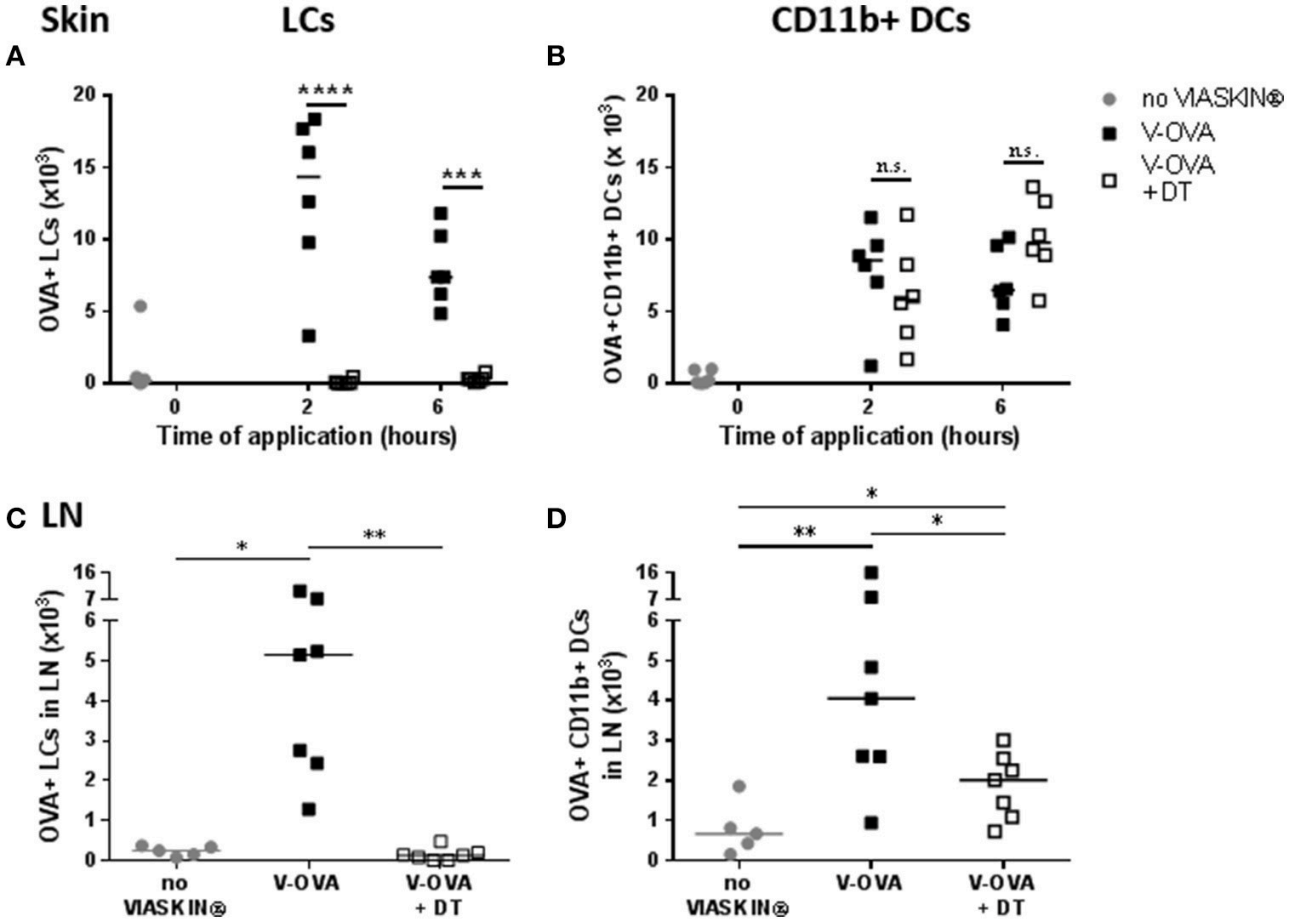
Effect of LCs depletion on allergen uptake by skin Dendritic Cells. VIASKIN®-OVA-AF647 (V-OVA) was applied on the back of sensitized mice (V-OVA) or sensitized mice after depletion of Langerhans cells (V-OVA + DT). Sensitized not treated mice were used as control (no VIASKIN®). Skin **(A,B)** were harvested after 2 or 6 h and sdLNs were harvested after 48 h. Cells were isolated and counted before immunostaining for flow cytometry analysis. In live single cells gate, migrating DCs were gated as MHC-II^high^ CD11c^+^ and LCs **(A,C)** and CD11b^+^ DCs **(B,D)** were identified as CD11b^+^EPCAM^+^XCR1^−^ and CD11b^+^EPCAM^−^XCR1^−^, respectively. Proportion of OVA^+^ cells inside each population were measured and reported to number of cells. Each symbol represents a mice and bar represent median. Experiment was reproduced twice independently. ^*^p < 0.05; ^**^p < 0.01; ^***^p < 0.001; ^****^p < 0.0001 by Kruskal-Wallis test followed by Dunn's multiple comparisons test.

To validate the possible role of LCs in the induction of tolerance by EPIT, we compared allergen uptake in sensitized LANG-DTR mice after depletion of LCs. After DT injection, depletion of LCs was confirmed by flow cytometry. As expected, no OVA^+^ LCs was detected following application of VIASKIN-OVA-AF647 in the skin of LC-depleted mice (Figure 5A). DT injection did not alter the CD11b^+^ DCs subset and allergen capture by those cells was no different in the skin of mice not LC-depleted after 2 or 6 h (Figure 5B).

In sdLN, no OVA^+^ LCs were detected, as expected (Figure 5C). Interestingly, LC depletion significantly decreased OVA^+^ CD11b^+^ DCs migration into sdLNs after 48 h (Figure 5D) even though antigen capture in the skin was not altered. This suggested a possible interaction between LCs and dermal DCs in the skin, that would be needed for CD11b^+^ DCs migration.

### Langerhans cells are required for the induction of regulatory T cells *in vivo*

**Figure 6:**
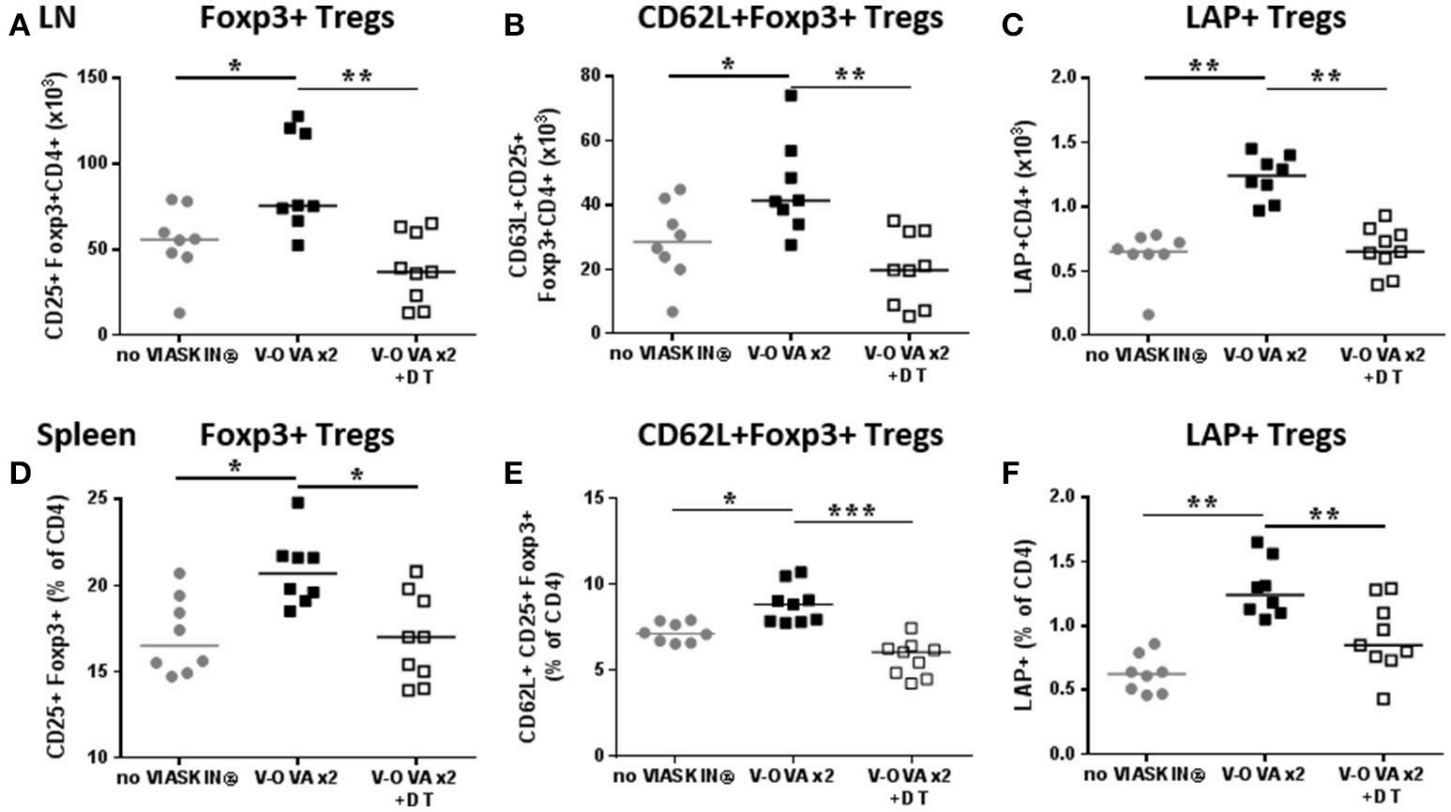
Short term induction of Tregs by EPIT requires LCs. VIASKIN®-OVA (V-OVA) was applied on the back of sensitized mice (V-OVA) or sensitized mice after depletion of Langerhans cells (V-OVA + DT) for 48 h once a week for 2 weeks and level of Tregs was evaluated the week after the 2nd application in sdLN **(A–C)** and spleen **(D–F)**. Sensitized not treated mice were used as control (no VIASKIN®). Cells were isolated and counted before immunostaining for flow cytometry analysis. Number of CD4^+^CD25^+^Foxp3^+^ **(A)**, CD4^+^CD62L^+^CD25^+^Foxp3^+^ **(B)** and CD4^+^LAP^+^ **(C)** cells in sdLN and proportion of CD25^+^Foxp3^+^ **(D)**, CD62L^+^CD25^+^Foxp3^+^ **(E)** and LAP^+^ **(F)** among CD4 cells were analyzed. Each symbol represents a mice and bar represent median. Experiment was reproduced twice independently. ^*^p < 0.05; ^**^p < 0.01; ^***^p < 0.001 by Kruskal-Wallis test followed by Dunn's multiple comparisons test.

Two weeks' treatment of sensitized mice with EPIT induced a local increase in regulatory T cells. The amount of CD25^+^Foxp3^+^ Tregs significantly increased in sdLNs of EPIT treated mice compared to untreated animals (Figure 6A). More specifically, the CD62L^+^ subset of Foxp3 Tregs was induced (Figure 6B). EPIT also induced greater numbers of LAP^+^ Tregs compared to untreated mice (Figure 6C). This induction of Tregs was not limited to local LNs but was also observed in spleen of treated mice compared no untreated animals (Figures 6D–F). In the absence of LCs, no induction of Tregs was observed either in sdLNs or in the spleen (Figure 6) after 2 weeks of treatment.

**Figure 7:**
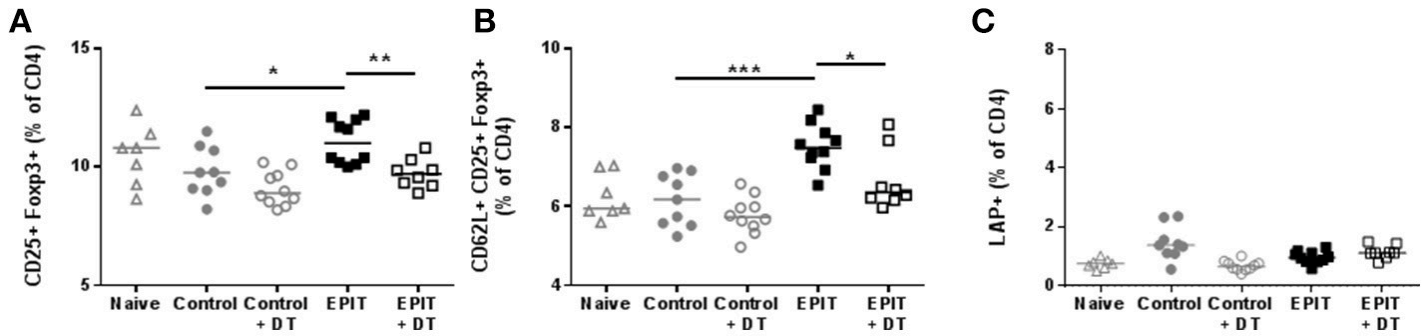
LCs are required for the induction of regulatory T cells. Sensitized mice were treated with VIASKIN® containing 100 μg OVA for 48 h once a week in presence (EPIT) or absence (EPIT + DT) of LCs. Not treated sensitized mice were used as control and effect of DT was also checked in a control group (Control + DT). After 8 weeks of treatment, induction of Tregs was measured by the proportion of CD25^+^Foxp3^+^ **(A)** and CD62L^+^CD25^+^Foxp3^+^ **(B)** and LAP^+^ **(C)** cells among CD4^+^ splenocytes Each symbol represents a mice and bar represent median. Experiment was reproduced twice independently. ^*^p < 0.05; ^**^p < 0.01; ^***^p < 0.001 by Kruskal-Wallis test followed by Dunn's multiple comparisons test.

Since the usual treatment period for EPIT in sensitized mice is 8 weeks, we checked the induction of Tregs in the spleen after 8 weeks of EPIT. We confirmed that DT alone did not alter normal responses as the Control + DT group showed similar levels of CD25^+^ Foxp3^+^ Tregs and LAP^+^ Tregs, and similar expression of CD62L (Figure 7). As expected, EPIT-treated mice had significantly higher levels of CD25^+^ Foxp3^+^ Tregs, especially CD62L^+^ Tregs compared to the Control group (Figures 7A,B). In contrast to mice treated for 2 weeks, no induction of LAP^+^ Tregs was observed after 8 weeks of treatment suggesting that the induction of LAP^+^ cells is transient. In the absence of LCs, no induction of Tregs was observed even after 8 weeks of EPIT treatment, confirming the crucial role of LCs in induction of Tregs by EPIT.

### Role of LCs in the induction of tolerance by EPIT

**Figure 8:**
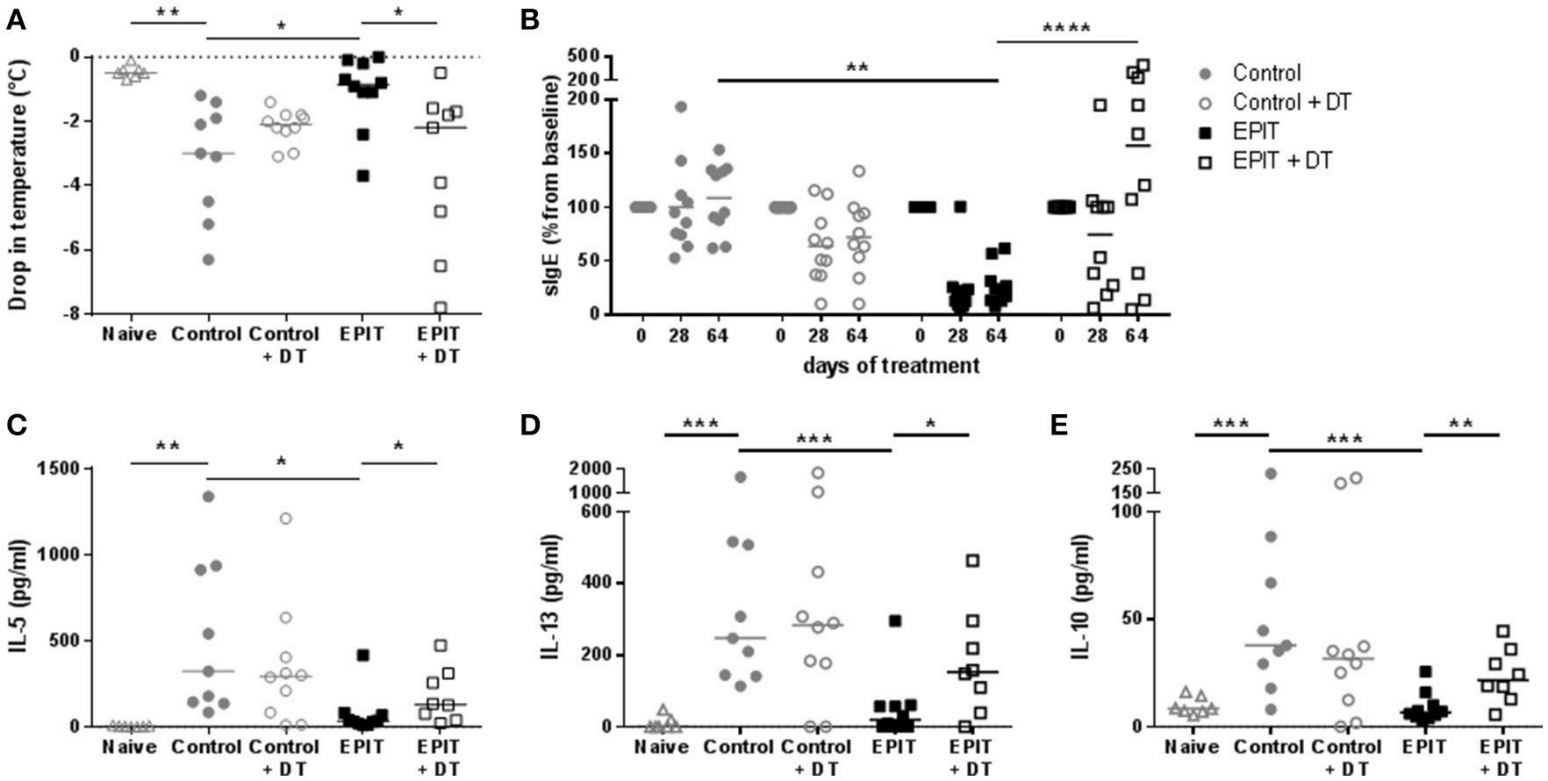
Desensitization induced by EPIT is not observed in absence of LCs. Sensitized mice were treated with VIASKIN® containing 100 μg OVA for 48 h once a week in presence (EPIT) or absence (EPIT + DT) of LCs. Not treated sensitized mice were used as control and effect of DT was also checked in a control group (Control + DT). After 8 weeks, mice were challenged orally and anaphylactic reaction was measured by the drop in temperatures **(A)**. Percentage of OVA-specific IgE compared to baseline was measured after 28 and 64 days of treatment **(B)**. Production of IL-5 **(C)**, IL-13 **(D)**, and IL-10 **(E)** by *in vitro* restimulated splenocytes isolated at the end of treatment were analyzed. Each symbol represents a mice and bar represent median. Experiment was reproduced twice independently. ^*^p < 0.05; ^**^p < 0.01; ^***^p < 0.001 by Kruskal-Wallis test followed by Dunn's multiple comparisons test **(A,C–E)**; and ^**^p < 0.01; ^****^p < 0.0001 by 2-way ANOVA followed by Tukey's multiple comparison test **(B)**.

To confirm that the induction of Tregs by EPIT is associated with desensitization, the anaphylactic response following oral challenge was evaluated in sensitized mice treated by EPIT and compared to untreated animals. Untreated mice experienced a significant decrease in body temperature (median = −3°C) following oral challenge with OVA compared to naïve mice. Injection of DT did not alter this response. EPIT treated mice showed a −0.85°C drop in body temperature, significantly less than that in untreated mice (Figure 8A). This protection from anaphylaxis was associated with a significant decrease of OVA-specific IgE levels (Figure 8B) and a decrease in cytokine response by *in vitro* re-stimulated splenocytes (Figures 8C–E) from EPIT-treated mice compared to untreated controls.

In mice treated in absence of LCs, oral challenge induced anaphylactic reactions with a significant drop in body temperature of −2.2°C compared to EPIT-treated mice (Figure 8A). EPIT did not induce any decrease of sIgE nor OVA specific cytokine responses when LCS were depleted (Figures 8B–E). These results support the crucial role of LCs in mechanisms of induction of tolerance by EPIT in sensitized mice.

## Discussion

The different populations of skin DCs are key players in antigen-specific immune activation or regulation. Indeed, skin has been proposed as route of sensitization, but perhaps more importantly, is also a way to induce tolerance, as in the case of EPIT. The role of the different population of skin DCs in this tolerance induction requires further clarification. In this study, we demonstrated that both LCs and CD11b^+^ dermal cDC2s can take up allergen in the skin and migrate to draining LN to induce regulatory T cells. However, the absence of LCs during EPIT impaired allergen uptake and Foxp3^+^ Treg induction, especially CD62L^+^ Tregs, resulting in absence of desensitization and protection from oral allergen exposure, indicating their crucial role in skin-induced tolerance.

The role of LCs in inducing allergic responses or suppressing immunity is still controversial. In the steady state, LCs have been shown to induce tolerance to skin antigen (16–18). LCs have also been implicated in Treg expansion in response to antibody-targeted antigen or irradiation (19, 20). Initiation of epicutaneous sensitization with protein antigens depended on LCs (21) whereas skin sensitization to house dust mite depended on dermal cDC2 and was even increased in absence of LCs (22). In our model, we demonstrated that LCs are critical for the induction of Tregs and subsequent desensitization of mice by EPIT, suggesting of tolerogenic capacities of LCs in the context of previously established sensitization. Differences of immunological status and/or keratinocyte activation could explain the divergence concerning the role of LCs between the different models, as we previously observed their impact on allergen uptake (12, 23).

Dermal cDC2s have been previously implicated in IL-17-mediated psoriasis-like response (24). These cells appear critical for the induction of Th2 responses and epicutaneous sensitization to house dust mite (22, 25). On the other hand, cDC2s can suppress antibody production by blocking the development of follicular helper T cells, and inducing Foxp3^+^ Tregs (26, 27). In our model, dermal CD11b^+^ cDC2s were as efficient as LCs in taking up allergen following epicutaneous application and migrating into sdLNs. Moreover, migrating cDC2s could induce Foxp3^+^ Tregs, suggesting that they may play a role in tolerance induction during EPIT. However, these cells are not sufficient since depletion of LCs without alteration of the CD11b^+^ DC compartment failed to induce Tregs *in vivo*. We can postulate that cDC2s play a role in the mechanisms of induction of Tregs in sensitized mice in which they need interaction of LCs to be fully activated and to migrate. Indeed, depletion of LCs did not alter allergen capture by skin cDC2s, but did impair their migration into sdLNs. Interaction between LCs and dermal DCs may be needed for optimal migration of dermal DCs. Cooperation of LCs and dermal DCs has been observed previously in the induction of Th2 responses (28), and migrating LCs can also interact with DCs to induce DC maturation and antigen transfer (29). Further studies are required to decipher the possible interactions between these populations and by which mechanisms LCs cooperate with DCs.

In addition to the possible interactions between LCs and dermal DCs, cDC2s were not sufficient to induce Tregs and provide protection *in vivo* because particular Treg populations implicated in EPIT were not induced by cDC2s. *In vitro*, migrating cDC2s induced Foxp3^+^ Tregs, but low levels of LAP^+^ Tregs. The induction of LAP^+^ Tregs has been observed during EPIT in different models (6, 9). The absence of induction of these LAP^+^ Tregs by CD11b^+^ DCs could explained the absence of protection observed after depletion of LCs. Noteworthy, the induction of LAP^+^ Tregs seems transient in this model with a significant increase following 2 weeks of treatment, which is no longer observed after 8 weeks of treatment either in the sdLN or in the spleen. The LAP^+^ Tregs may participate in the first mechanistic steps of EPIT to induced CD25^+^Foxp3^+^ Tregs that are crucial for efficacy of EPIT (8).

One unique feature of EPIT is its ability to induce both CD62L^+^ and CD62L^−^ Foxp3^+^ Tregs, whereas only CD62L^−^ Tregs are induced by other immunotherapies (oral and sublingual) in our models (9). This population of CD62L^+^ Tregs seems central to the mechanism of EPIT given the epigenetic modification, i.e., foxp3 demethylation, the sustained protection and the bystander effect, which were recently shown to be features of the CD62L^+^ population of Tregs (10). Migrating LCs induced significantly more CD62L^+^ Tregs than cDC2s *in vitro* and the depletion of LCs abrogated any induction of CD62L^+^ Tregs *in vivo*. Given the importance of this particular population of Tregs in EPIT, the capacity of LCs to induced them may explain their crucial role in efficacy of EPIT.

As previously shown for gut DCs (14, 15), skin migrating LCs, but not cDC2s, expressed higher level of integrin beta8, suggesting that requirement of LCs to induce particular population of Tregs could be due to their capacity to activate TGF-β. However, further experiments are needed to decipher the exact role of this pathway in mechanism of induction of Tregs during EPIT.

This unique property of cutaneous LCs is also distinguished from the induction of Tregs with sublingual immunotherapy (SLIT). Although Langerhans-like cells are present in the oral mucosa, studies of allergen uptake during SLIT in an animal model demonstrated an important role for both CD11b^+^ DCs and CD11b^+^ macrophage-like cells (30, 31). These populations of APCs support the differentiation of Tr1 cells. Thus, the difference of Tregs induced by EPIT compared to SLIT may be explained by the intrinsic difference of APC implicated in these different routes of treatment. Similarly, subcutaneous immunotherapy, which “bypasses” the epidermidis and resident LCs, induces Tr1 cells, suggesting the importance of LCs, and epicutaneous route for induction of the CD62L^+^ population of Tregs.

In conclusion, we demonstrated that during EPIT of sensitized mice, allergen is taken up by LCs and dermal cDC2s that migrate to the sdLNs to induce Tregs. However, the capacity to induce both CD62L^+^ Foxp3^+^ Tregs and LAP+ Tregs reside mainly in LCs. Indeed, the absence of LCs during EPIT decreased treatment efficacy indicating their crucial role in skin-induced tolerance.

## Ethics statement

Project license from French authorities #7811.

Comité d'Ethique en Expérimentation Animale de DBV Technologies N°127 (CEEA127).

## Author contributions

ViD wrote the article and is corresponding author. LM had valuable contribution to the underlying concept and the design of the research. LL, VéD, CP, and AB performed the experiments and some analysis. CD and HS (senior author) had valuable contribution to the underlying concept and the design of the research.

### Conflict of interest statement

This study was supported by DBV Technologies, the developer and owner of Viaskins. ViD, LM, LL, VéD, CP, AB, HS are DBV Technologies employees. CD received honoraria and/or compensation with regard to the study, as investigators, coordinators or experts, in relation with the time spent on the study.
